# The mechanisms underlying COVID-19 induced insulin resistance: a narrative review

**DOI:** 10.3389/fendo.2026.1781679

**Published:** 2026-03-11

**Authors:** Bing Zhu, Shen Qu, Jue Li, Wei Deng, Wen-Jun Shen, Jia Chen

**Affiliations:** 1 Department of Endocrinology and Metabolism, Shanghai Public Health Clinical Center, Fudan University, Shanghai, China; 2 Department of Endocrinology and Metabolism, Shanghai Tenth People’s Hospital, Institute of Obesity, Tongji University School of Medicine, Shanghai, China; 3 SinoUnited Health Endocrinology, Metabolism & Thyroid Center, Shanghai, China; 4 Shanghai Tongji Hospital, Tongji University School of Medicine, Shanghai, China; 5 Department of Endocrinology, Beijing Jishuitan Hospital, Capital Medical University, Beijing, China; 6 Department of Medicine, Stanford University School of Medicine, Stanford, CA, United States

**Keywords:** COVID - 19, immune system, inflammatory, insulin resistance, lifestyle change, long covid, mechanism, metabolism

## Abstract

The COVID-19 pandemic, caused by SARS-CoV-2, has resulted in a significant increase in insulin resistance and new-onset diabetes among recovered individuals. This review examines the multifactorial mechanisms underlying these metabolic complications, including activation of the immune system and inflammatory cascades, lifestyle changes, nutritional deficiencies, imbalances in amino acid metabolism, alterations in ketogenesis, disruptions in the gut microbiome, psychological impacts, and COVID-19 vaccines. We discuss how these factors collectively contribute to insulin resistance, particularly in the context of COVID-19, and highlight potential therapeutic strategies, such as dietary interventions and ACE2 activators, that may mitigate these effects. Our analysis underscores the need for targeted approaches to prevent and treat insulin resistance in post-COVID-19 patients, emphasizing the importance of understanding the pandemic’s long-term metabolic consequences.

## Introduction

During the most recent pandemic, the Coronavirus disease 2019 (COVID-19), caused by severe acute respiratory syndrome coronavirus 2 (SARS-CoV-2), had spread to majority of countries, areas, or territories worldwide. According to the World Health Organization, there were over 778 million infection cases and more than 7,074,400 confirmed deaths (https://data.who.int/dashboards/covid19/cases?n=c). The continued rise in cases remains to be concerning for the public. Many patients have persistent symptoms for more than four weeks and developed ‘long COVID’ (1–3). It includes both ongoing symptomatic COVID-19 (from 4 to 12 weeks) and post-COVID-19 syndrome (12 weeks or more) (3).

Diabetes has emerged as one of the long-term sequelae of COVID-19. Reports suggest that approximately 4.2% of recovered COVID-19 individuals are newly diagnosed with diabetes (4). A cross-sectional analytical study conducted at a Tunisian referral center revealed that the incidence of new-onset diabetic ketoacidosis (DKA) surged by 48.17% during the COVID-19 pandemic (March 2020–January 2022) relative to the pre-pandemic period (March 2018–March 2020), with the incidence of type 1 diabetes mellitus (T1DM) and type 2 diabetes mellitus (T2DM) increasing by 50% and 44%, respectively (5). However, the risk of new-onset diabetes following COVID-19 infection is dependent on the duration of follow-up. In a UK-based study, the risk of new-onset diabetes in individuals with COVID-19 was 81% at 5–12 weeks post-infection, but it diminished to 7% at 13–52 weeks (6). A multi-country investigation indicated that the risk of new-onset diabetes was 26% at 30–89 days after COVID-19 infection, which subsequently decreased to 11% at ≥90 days (7). In a US study, Wander et al. found that men with COVID-19 had a 156% higher risk of new-onset diabetes within 120 days of infection compared to those without COVID-19, but this risk reduced to 95% over the entire follow-up period of 456 days (8). Nevertheless, Montefusco et al. demonstrated that individuals who develop COVID-19-associated new-onset hyperglycemia are more likely to experience persistent rather than transient hyperglycemia (9). Thus, it is imperative and critical to concentrate on preventing the progression to new-onset diabetes in high-risk individuals during the critical 3–6-month window following SARS-CoV-2 infection.

**Figure 1:**
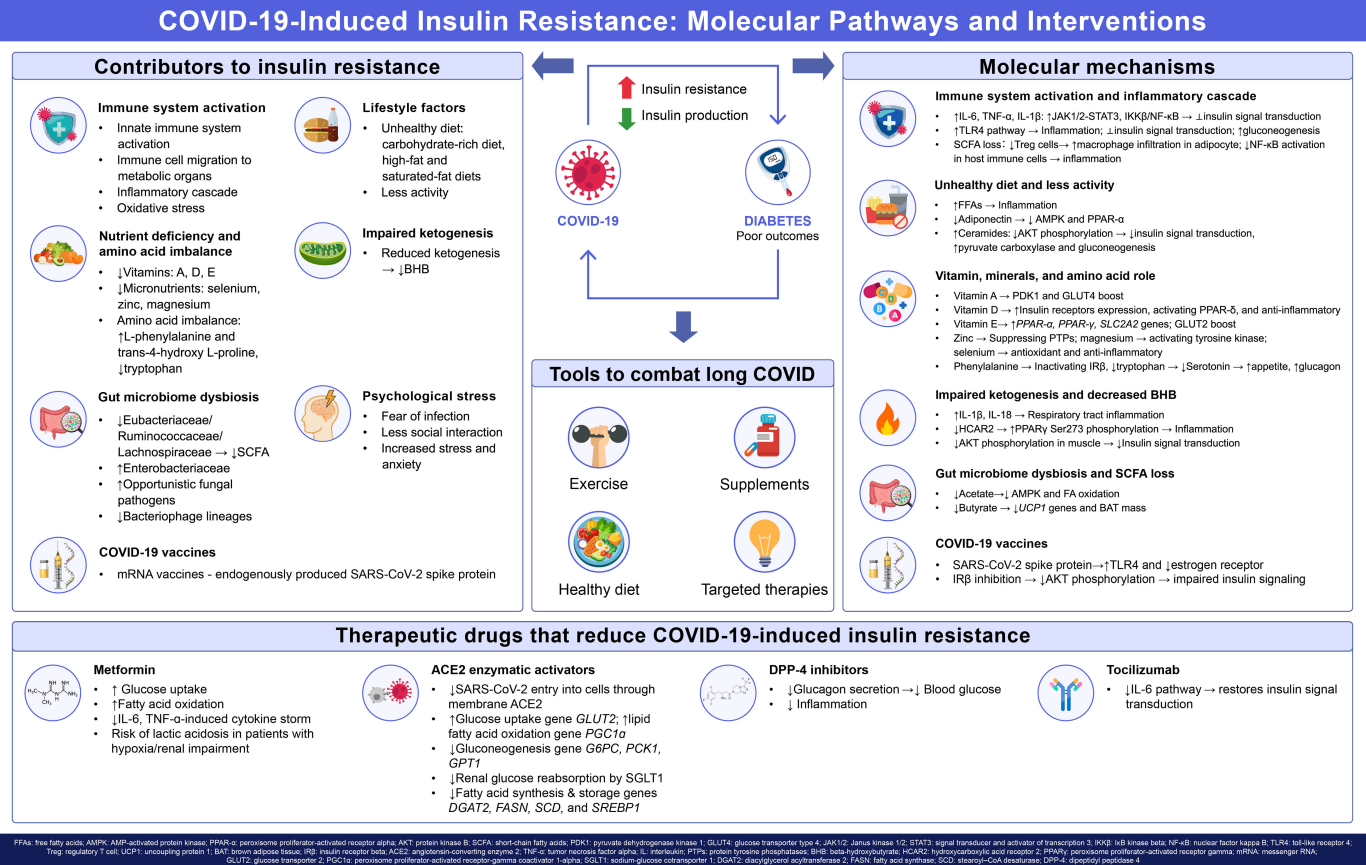
Contributors, molecular mechanisms and interventions of COVID-19-induced insulin resistance. SARS-CoV-2 infection leads to insulin resistance via multiple interconnected factors, including innate and adaptive immune activation-driven inflammatory cascades and oxidative stress, pandemic-associated unhealthy lifestyles, nutrient and amino acid imbalances, impaired ketogenesis, gut microbiome dysbiosis, psychological stress, and direct effects of SARS-CoV-2 spike protein from infection and vaccines on insulin signaling. Core molecular mechanisms involve inhibited insulin signal transduction, disrupted glucose and lipid metabolism such as enhanced gluconeogenesis and impaired AKT phosphorylation, and chronic inflammation. Interventions for long COVID-associated insulin resistance include lifestyle modifications, nutrient supplementation, microbiome modulation, and therapeutics drugs such as metformin, ACE2 enzymatic activators, which act by restoring insulin signaling, suppressing inflammation, regulating glucose metabolism and inhibiting viral entry.

The pathogenesis of new-onset diabetes in COVID-19 patients is multifactorial and intricate, primarily involving pancreatic β-cell dysfunction and insulin resistance. Angiotensin-converting enzyme 2 (ACE2), the principal receptor for SARS-CoV-2, is highly expressed in both the exocrine and endocrine pancreas, as evidenced by high mRNA levels (10). Indeed, Muller et al. further confirmed that SARS-CoV-2 can infect human pancreatic cells, both ex vivo and *in vivo* (11). In another study, healthy human islets were isolated and infected with SARS-CoV-2 ex vivo. The results revealed that β-cells underwent apoptosis and exhibited reduced insulin secretion following SARS-CoV-2 infection (12). In our previous clinical study, we observed that SARS-CoV-2 infection decreased fasting insulin secretion in patients without a prior history of diabetes at the acute phase of COVID-19 (13). However, this effect appeared to be transient and reversible. We demonstrated that the fasting C-peptide levels and HOMA-CP had significantly increased at 3-month post COVID-19 and remained unchanged at the 6-month follow-up (13). Therefore, pancreatic islet dysfunction is more likely to be responsible for the hyperglycemia during the acute phase of COVID-19 (up to 4 weeks). In contrast, hyperglycemia in long COVID may be attributed to insulin resistance. Our previous research has laid the foundation for this conclusion. We demonstrated that SARS-CoV-2 infections exacerbate insulin resistance, which persists at both the 3-month and 6-month follow-up in individuals without known diabetes (13). Similarly, Montefusco et al. also demonstrated the presence of insulin resistance in patients with COVID-19 without a prior history of diabetes and suggested the persistence of aberrant glycometabolic control long after the recovery from the disease (9). Indeed, extensive clinical datasets indicate that both acute and chronic COVID-19 infection favor insulin resistance, thereby posing a risk for individuals with pre-diabetes to develop T2DM (14). However, the potential mechanisms driving insulin resistance in individuals who had COVID-19 remain unclear. Cao et al. conducted genetic correlation and Mendelian randomization analyses to investigate the genetic relationships between T2DM and the severity of COVID-19, their results suggest that susceptibility to COVID-19 may increase the risk for T2DM (15). The purpose of this review is to summarize the potential mechanisms aside from genetic factors by which the COVID-19 pandemic may trigger insulin resistance. **Figure 1** summarizes the contributors, molecular mechanisms, and interventions of COVID-19-induced insulin resistance in the form of a graphical abstract.

## Method

The literature search for this narrative review was conducted across electronic databases, including PubMed and Web of Science, with no restrictions on the initial publication year. The key search terms included combinations of “COVID-19”, “SARS-CoV-2”, “long COVID”, “insulin resistance”, “insulin signaling pathway”, “pathogenesis”, and the associated content keywords for each independent chapter.

*Inclusion criteria*: oiginal research articles, review papers, case-control studies, cohort studies, *in vitro* experiments, and animal models that explicitly investigate the mechanisms linking COVID-19 to insulin resistance were included. Studies exploring related metabolic perturbations with direct implications for insulin sensitivity in non-COVID-19 population were also included. All research included has complete abstracts or full-text access, which allows for rigorous evaluation of methodological quality and findings. *Exclusion criteria*: Case reports, letters to the editor, conference abstracts, and unpublished data with insufficient detail on study design, sample size, or mechanistic endpoints were excluded.

Following the initial search, two independent reviewers screened titles and abstracts to identify eligible studies. Full-text articles were retrieved for further evaluation, and discrepancies between reviewers were resolved through consensus or consultation with a third senior researcher. The final selection of literature was based on the relevance to the review’s core theme, methodological rigor, and contribution to elucidating the underlying mechanisms of COVID-19-induced insulin resistance.

## Involvement of immune system in COVID-19 and insulin resistance

**Figure 2:**
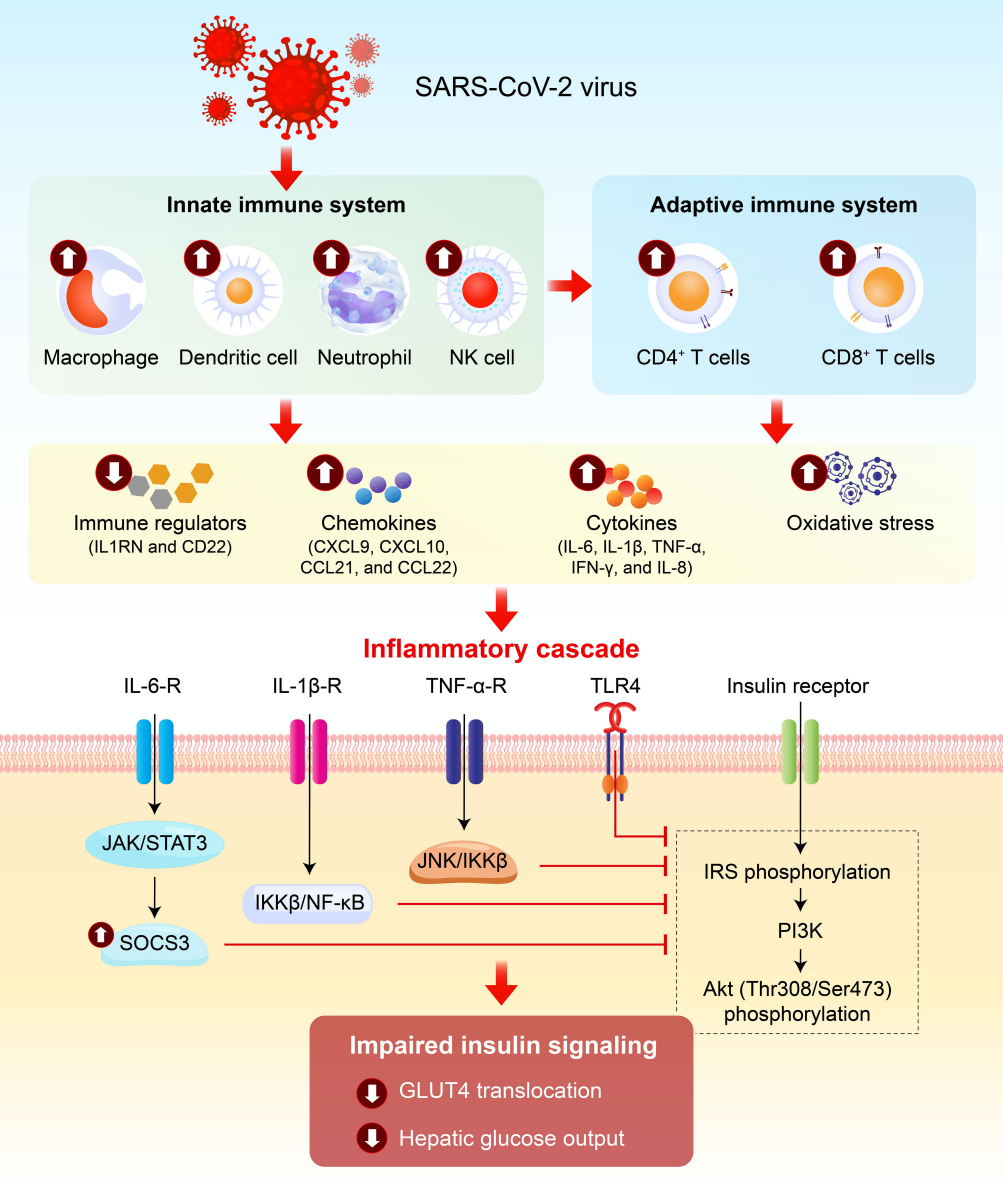
Immune-inflammatory signaling pathways linking COVID-19 to insulin resistance. SARS-CoV-2 infection dysregulates both innate and adaptive immune systems, triggering sustained production of pro-inflammatory cytokines (IL-6, IL-1β, TNF-α, IFN-γ) and chemokines, alongside elevated immune regulatory proteins and oxidative stress in COVID-19 patients. These factors drive an inflammatory cascade that impairs insulin signaling: IL-6 activates JAK1/2-STAT3/SOCS3, IL-1β and TNF-α trigger IKKβ/NF-κB and JNK pathways, and SARS-CoV-2 spike protein binds TLR4-all inducing inhibitory serine phosphorylation of IRS proteins, reducing PI3K/Akt activation, and impairing GLUT4 translocation and hepatic glucose output. This immune-inflammatory axis is a central molecular mechanism linking COVID-19 to persistent insulin resistance.

Immune system is one of the first to react to the COVID-19 infection and both the innate and adaptive immune systems may suffer from the infection of SARS-CoV-2 with unique acute and prolonged residual effects. **Figure 2** depicts the involvement of immune system and inflammatory cascade in COVID-19 and insulin resistance.

### Unique immunological dysfunction caused by COVID-19

Both the innate and adaptive immune systems may suffer from the infection of SARS-CoV-2 with unique acute and prolonged residual effects. A growing number of studies indicate that even mild-to-moderate acute COVID-19 can lead to ongoing and sustained immunological dysfunction and inflammatory response, which are distinct from those following infections with other coronaviruses (16, 17). For instance, Yin et al. studied the serology and used “omic” analysis of the blood samples from individuals with long COVID eight months post infection (17). They found that long COVID patients had higher levels of SARS-CoV-2-specific CD8+ T cells than recovered individuals. Additionally, long COVID patients exhibited increased IL-6 expression in CD4+ T cells and higher frequencies of CD4+ T cells primed to migrate to inflamed tissues. Furthermore, Olink proteomics analysis revealed that proteins linked to immune regulation (IL1RN and CD22) and inflammation (LGALS9, CCL21, CCL22, TNF, CXCL10 and CD48) are increased in Long COVID individuals compared to those who had recovered.

### Production of pro-inflammatory cytokine

COVID-19 triggers immune cell migration to key metabolic organs, leading to increased production of pro-inflammatory cytokines (e.g., IL-6, TNF-α, IL-1β) produced by macrophages and monocytes (18). This phenomenon appears not to be restricted to individuals in the acute phase of COVID-19 infection. For example, Montefusco et al. demonstrated elevated serum levels of various pro-inflammatory cytokines, including IL-1β, IL-8, IL-17, G-CSF, and IFN-γ, in individuals who had recovered from COVID-19 (9). In a similar vein, Phetsouphanh et al. reported a set of pro-inflammatory cytokines (interferon β (IFN-β), PTX3, IFN-γ, IL-6, IL-8, CXCL9, CXCL10), the elevation of which are highly associated with Long COVID (16). Additionally, Schultheiß et al. found that individuals with Post-Acute Sequelae of COVID-19 (PASC) exhibited persistent elevated levels of IL-1β, IL-6, and TNF in a gender-independent manner, with no clear time-dependent decrease. They suggested that the persistent elevation of these three cytokines may serve as a hallmark of long COVID. Moreover, this study further revealed that this trio of cytokines is specifically produced by the myeloid/macrophage population in the lungs, as well as by pro-inflammatory macrophages derived from bronchoalveolar lavage fluid (19).

### Immune dysregulation and inflammation-related insulin resistance

The immune dysregulation and cytokine storm during the acute phase of COVID-19 rapidly impair insulin sensitivity. Since these pathological processes can persist long-term even after recovery, inflammation-related insulin resistance may continue and potentially worsen gradually post-COVID-19. These processes can impair insulin sensitivity through:

1. Inflammatory cascade: IL-6 activates JAK1/2-STAT3 signaling, inducing SOCS3 expression and serine phosphorylation of IRS (inhibitory), thereby blocking insulin signal transduction (20). Furthermore, IL-1β activates IKKβ/nuclear factor kappa-light-chain-enhancer of activated B cells (NF-κB) pathway and increases the phosphorylation of IRS-1 at Ser307, inhibiting insulin signaling cascade (21). Moreover, acute elevation of TNF-α can activate JNK and IKKβ in the liver, thereby inducing serine phosphorylation of IRS-1 and subsequently inhibiting the insulin-induced activation of the IRS-1/PI3K/Akt pathway (22).
2. TLR4 (Toll-like receptor 4) activation: The SARS-CoV-2 S protein is shown to be able to bind to TLR4 with a high affinity (23). Additionally, COVID-19 increases gut permeability (24), allowing endotoxins to activate TLR4 and trigger the activation of the TLR4/MD2 complex through heterodimerization, and result in recruitment of the MyD88 and TRIF, which increase the production of pro-inflammatory mediators recruiting more downstream participants (25). Furthermore, pro-inflammatory cytokines promote FFAs release from adipocytes (21), activating TLR4 signaling and suppressing insulin signaling in skeletal muscle and adipocyte (26).
3. Oxidative stress: Viral-specific CD8+ T cells can trigger T cell-mediated liver inflammation by activating Kupffer cells (liver-resident macrophages) (27). Additionally, the endocytosis of TLR4-MD2 complex by hepatic macrophages can generate reactive oxygen species (ROS) through NADPH oxidase 2 (28). Excessive ROS production leads to oxidative stress, impairs insulin receptor signaling through activation of JNK, p38 mitogen-activated protein kinase (MAPK), IKKβ, NF-κB, and PKC (29, 30), and impairs insulin-induced GLUT4 translocation in adipocytes (31).
4. Enhanced hepatic gluconeogenesis: During stress, IL-6 activates the STAT3 signaling pathway to induce hepatic metabolic reprogramming, significantly upregulating the expression of *PEPCK* and *G6Pase*, thereby promoting gluconeogenesis (32).

## Lifestyle changes in COVID-19 and implications for insulin resistance

Social distancing was one of the most widely adopted health policies during the COVID-19 pandemic. These restrictions led to significant changes in people’s lifestyles and daily behaviors (33, 34). Studies have reported that some individuals developed unhealthy habits, including reduced physical activity, less social interaction, increased stress and anxiety, emotional eating, and increased consumption of unhealthy foods, such as high-fat and saturated-fat diets (35, 36). According to a survey in an Italian center, more than half of the respondents (67.2%) indicated that they had consumed more bread, pasta, and pizza while under lockdown (33). Another Italian survey involving 41 obese children aged 6 to 18 years found that the intake of potato chips, red meat, and sugary drinks surged significantly during the lockdown (34). Similarly, In Palestine, 31.5% of adolescents reported consuming more sugar-added drinks, 36.7% said they ate more fried foods, and 46.5% indicated an increase in their consumption of sweets and sugar-added foods compared to before the lockdown. In terms of physical activities, data from an experimental longitudinal study involving 2,426 children and adolescents across five schools in Shanghai revealed a significant decline in the median time spent on physical activities. It dropped from 540 minutes per week before the pandemic to 105 minutes per week during the pandemic (37). Such lifestyle changes are reflected in an increased risk of obesity. A retrospective cohort study carried out at the Growth Clinic of Seoul St. Mary’s Hospital, involving school-aged children aged 4 to 14 years, revealed that the proportion of overweight or obese children increased from 23.9% in the pre-COVID-19 period to 31.4% during the COVID-19 period (38).

In the presence of unhealthy diets and a positive energy balance, visceral and subcutaneous adipose tissues expand in ways that are largely genetically determined (39). This expansion leads to increased free fatty acids (FFAs), low-grade inflammation, and dysregulation of hepatokine and adipokine production, thus promoting insulin resistance. For example, a carbohydrate-rich diet has been shown to decrease plasma adiponectin levels (40). Adiponectin possesses anti-inflammatory, antioxidative, and insulin-sensitizing properties (41). A reduction in adiponectin levels may lead to the development of obesity-related insulin resistance (42). In terms of mechanism, adiponectin can stimulate AMP-activated protein kinase (AMPK) phosphorylation and activation (43), increasing fatty acid oxidation and energy expenditure, partly through PPARα activation (44) in the liver and skeletal muscle. This process enhances insulin sensitivity *in vivo*. Indeed, when compared to individuals without COVID-19, patients with COVID-19 had lower adiponectin levels, independent of sex (45). Leptin, another adipokine primarily secreted by white adipose tissue, increases in response to chronic overnutrition (46). Hyperleptinemia has also been identified as a clinical feature in COVID-19 patients (45). A partial reduction in plasma leptin levels has been shown to reduce weight gain and improve insulin sensitivity in the context of obesity (47).

## Vitamins and microelements deficiency and implications for insulin resistance

COVID-19 patients may consume fewer fresh vegetables and fruits due to isolation (33). Additionally, they may experience symptoms such as loss of appetite and impaired digestion and absorption. Previous study has indicated that 76% of hospitalized exhibit at least one persistent symptom for a minimum of 6 months following SARS-CoV-2 infection (48), with intestinal manifestations such as loss of appetite (24%), nausea (18%), and diarrhea (15%) being reported in patients with long COVID (49). These symptoms can lead to insufficient intake and utilization of essential nutrients, including vitamins and microelements as illustrated in **Table 1**. An observational study has shown a decreased plasma levels of Vitamin A, Vitamin D, Vitamin E, Zinc, and Selenium in patients hospitalized for COVID-19 during the first wave of the pandemic (50).

**Table 1:** Vitamins and Microelements Deficiency and Implications for Insulin Resistance in COVID-19.

Nutrients	Role in insulin signaling pathway	Levels in COVID-19 patients	Clinical implications
Vitamin A	• Retinoic acid (RA) acts on RAR and PPAR-δ, upregulating insulin signaling genes (PDK1, GLUT4) • Beneficial for lipid and energy homeostasis	Decreased	Promote insulin resistance
Vitamin D	• Involved in insulin receptor transcription • 1,25(OH)_2_D enhances insulin receptor expression, activates PPAR-δ • Anti-inflammatory effects	Decreased	Promote insulin resistance
Vitamin E	• Upregulates PPAR-alpha, PPAR-gamma, and SLC2A2 gene expression • Downregulates CD36 gene expression	Decreased	Promote insulin resistance
Selenium	• Antioxidant and anti-inflammatory	Decreased	Promote insulin resistance
Zinc	• Regulates protein tyrosine phosphate activity in IGF-1 signaling • Stimulates glucose uptake and lipogenesis in adipocytes	Decreased	Promote insulin resistance

### Vitamin A

Recently, a cross-sectional study observing vitamin A in COVID-19 patients revealed reduced plasma levels in patients during acute inflammation (51). Vitamin A and its active metabolites, retinoic acid (RA), have demonstrated beneficial effects on lipid and energy homeostasis, with potential implications for metabolic diseases such as obesity and insulin resistance (52). RA acts as a ligand for the classical retinoic acid receptor (RAR) and peroxisome proliferator-activated receptor-delta (PPAR-δ) (53). In mature adipocytes, both RA and the PPAR-δ-specific synthetic ligand GW0742 have been shown to increase the expression of genes in insulin-signaling cascades, including PDK1 and the glucose transporter GLUT4. This improves insulin action and helps reduce obesity and insulin resistance (54, 55).

### Vitamin D

Carpagnano et al. reported an increased prevalence of vitamin D deficiency in COVID-19 patients (56). Similarly, a study involving 40 COVID-19 patients aged between 3 months and 18 years, as well as 45 healthy age-matched controls, demonstrated that children with COVID-19 had significantly lower vitamin D levels (13.14 μg/L) compared to the controls (34.81 μg/L) (57). A previous study identified a vitamin D response element in the promoter region of the insulin receptor gene, suggesting that vitamin D plays a role in the transcriptional regulation of insulin (58). The biologically active form of vitamin D, 1,25(OH)_2_D, has been shown to improve insulin sensitivity by enhancing the expression of insulin receptors and activating PPAR-δ (59). Additionally, vitamin D has anti-inflammatory properties, which can alleviate chronic inflammation and suppress inflammatory cytokines linked to insulin resistance (60). These findings align with clinical observations. Numerous meta-analyses and randomized controlled trials (RCTs) have demonstrated that vitamin D supplementation reduces homeostatic model assessment for insulin resistance (HOMA-IR), fasting insulin, and fasting blood glucose levels, while increasing the quantitative insulin-sensitivity check index (61, 62).

### Vitamin E

Vitamin E is a fat-soluble vitamin with antioxidant and anticoagulant properties that can neutralize free radicals (63). Although there still lasks data showing a direct benefitial effect of vitamin E in COVID-19 patient, recent meta-analyses and narrative reviews have suggested that vitamin E supplementation, or a vitamin-E-rich diet such as the Mediterranean diet, significantly lowers HbA1c, fasting insulin, and HOMA-IR in patients with T2DM (64, 65). An animal study found that vitamin E supplementation upregulated the expression of PPAR-alpha, PPAR-gamma, and SLC2A2 genes, while downregulating CD36 gene expression (66). PPAR-alpha is a key regulator of fatty acid oxidation pathways (67), and PPAR-gamma is a potent modulator of lipid metabolism and insulin sensitivity (67). SLC2A2 encodes the GLUT2 transporter, which is essential for glucose-mediated insulin secretion in humans (68). In contrast, upregulation of CD36, a fatty acid translocase, in the liver has been associated with insulin resistance in patients with fatty liver disease (69).

### Microelements

Several microelements, which decreased in patients hospitalized for COVID-19, play crucial roles in the insulin signaling pathway. Selenium, a key component of selenoproteins, has significant antioxidant and anti-inflammatory properties (70). Several randomized controlled trials (RCTs) have shown that selenium supplementation can reduce inflammation, alleviate oxidative stress, and improve insulin sensitivity (71). Zinc is also critical for insulin synthesis, secretion, and function. Similar to insulin, Zinc can also regulate protein tyrosine phosphate activity in insulin-like growth factor-1 (IGF-1) signaling, and in turn stimulate glucose uptake and lipogenesis in isolated adipocytes (72, 73).

However, it should be acknowledged that direct clinical evidence linking vitamin and trace element deficiencies to insulin resistance in post-COVID patients is insufficient. Most supporting evidence is extrapolated from non-COVID populations, which only indicates biological plausibility rather than definitive causality. Whether restoring these vitamins and micronutrients could ameliorate insulin resistance in post-COVID individuals remains to be confirmed, and the proposed mechanism should be regarded as hypothetical.

## Amino acid metabolism imbalance in COVID-19 and implications for insulin resistance

Recent research has highlighted an imbalance in amino acid metabolism in COVID-19 patients. Atila et al. compared the amino acid profiles of 30 amino acids between COVID-19 patients and healthy subjects and found significantly higher levels of serum trans-4-hydroxy L-proline and L-phenylalanine in COVID-19 patients (74). L-phenylalanine emerged as a key differential marker for COVID-19 patients. Interestingly, Zhou et al. challenged mice with a phenylalanine-rich and aspartame-containing diet and found that phenylalanine promoted the onset of T2DM in a dose-dependent manner. They also confirmed that serum phenylalanine levels in T2DM patients were higher and were positively correlated with HbA1c levels (75). Furthermore, the study demonstrated that phenylalanyl-tRNA synthetase (FARS) phenylalanylates lysine residues 1057 and 1079 on the insulin receptor beta (IRβ) by employing phenylalanine as a substrate, inactivating IRβ and leading to insulin resistance. It is well known that insulin signaling involves the recruitment of the insulin receptor substrate (IRS) proteins (76), which are activated via phosphorylated IRβ, leading to the promotion of GLUT4-mediated glucose uptake (77).

Previous studies have shown that acute COVID-19 patients and long COVID patients have reduced plasma tryptophan levels (78). Most circulating serotonin (5-hydroxytryptamine, 5-HT) is produced from dietary tryptophan in enterochromaffin cells in the gastrointestinal tract (79). Indeed, comparison of the metabolites in Long COVID patients and healthy controls revealed that serotonin was mostly depleted in individuals with PASC (80). Similarly, Wong et al. also confirmed that serotonin is the most significant among the molecules that were depleted in both acute and post-acute COVID-19 patients (81).

To investigate whether the reduction in serotonin levels in long COVID are due to unresolved inflammation caused by virus infection, Wong et al. recreated viral-induced inflammation in mice using double-stranded RNA poly(I:C) that were synthesized in the lab. As expected, they confirmed that viral RNA sensing and IFN induction via TLR3 result in depletion of serotonin. They also identified three mechanisms for this depletion: (1) Reduced intestinal absorption of tryptophan (due to a tryptophan-deficient diet and reduced tryptophan uptake caused by downregulation of *Ace2 and Slc6a19* genes); (2) decreased serotonin storage result from hyperactive platelets and thrombocytopenia; (3) Increased monoamine oxidase (MAO)-mediated serotonin turnover (81).

Serotonin is a key neurotransmitter involved in neural activity and neuropsychological processes and plays a crucial role in energy balance (82). Central serotonin suppresses appetite through 5-HT receptors (HTRs), primarily HTR_2_C, in neurons like proopiomelanocortin (POMC) (83) and dopaminergic neurons (84). Serotonin can also activate brown adipose tissue (BAT) and therefore, increases energy expenditure (85). In peripheral tissues, secretion regulates glucose metabolism: it activates α-cell HTR_1_F and inhibits glucagon secretion in the pancreas (86) and enhances glucose uptake in skeletal muscle by activating HTR_2_A (87). Together, reduced serotonin maybe involved in insulin resistance in long COVID patients.

## Ketogenesis and its role in COVID-19 and insulin resistance

Previous studies have demonstrated elevated serum β-hydroxybutyrate (BHB) levels in patients with influenza compared to healthy controls, suggesting that viral infections can induce ketogenesis. However, in contrast, patients with COVID-19 was shown to have reduced BHB concentrations, indicating that this virus may impair infection-induced ketogenesis (88).

Karagiannis et al. showed that BHB increased the number of CD4+ T cells and enhanced their production of interferon gamma (IFNγ) *in vitro*. This effect was reversed by knocking down the beta-hydroxybutyrate dehydrogenase 1 (BDH1) gene, which mediates ketolysis (88). Their data further suggest a link between impaired ketogenesis in SARS-CoV-2 infection and dysfunctional CD4+ T cell immunity. Additionally, Argüello et al. revealed that BHB enhanced mitochondrial function and improved the ability of CD4+ T cells to metabolize amino acids and fatty acids in the absence of glucose (89). Therefore, BHB may serve as a crucial energy source to maintain T cell function in nutrient-deprived microenvironments during severe viral infections.

Indeed, in the acute respiratory distress syndrome (ARDS) that are caused by COVID-19, BHB fuels into the tricarboxylic acid (TCA) cycle and cause the T cells to rely on oxidative phosphorylation (OXPHOS) while promoting the synthesis of amino acids that are drained during infection (90). Moreover, BHB has been shown to reduce inflammation in the respiratory tract (91, 92). Possible mechanisms include promoting autophagy (93), suppressing the mast cell/IL-2 axis (91), and reducing NLRP3 inflammasome-mediated increase of interleukin-18 (IL-18) and interleukin-1β (IL-1β) (92). These data suggest that ketogenesis may contribute to resistance against oxidative stress and inflammation-associated insulin resistance.

Recent studies further support the positive role of ketones in insulin sensitivity. Mey et al. (94) conducted a randomized crossover study to understand whether liver generates ketones to protect against insulin resistance. They produced a 12h overnight intravenous lipid infusion in nineteen healthy adults to provoke acute insulin resistance and used hyperinsulinemic–euglycemic clamps to measure insulin sensitivity for both hepatic and peripheral tissue. They observed a positive correlation between plasma BHB levels and insulin-stimulated suppression of hepatic glucose production, suggesting that hepatic ketogenesis may facilitate hepatic insulin sensitization. Similarly, Myette-Cote et al. (95) recruited twenty healthy participants and conducted a randomized cross-over study, where participants took ketone monoester supplements, and the changes in insulin concentrations and estimates of insulin sensitivity were documented. They demonstrated that exogenous ketone supplementation improved insulin sensitivity and reduced blood glucose levels.

The effectiveness of ketogenic diets in lowering fasting blood glucose and HbA1c and ameliorating insulin resistance have also been confirmed in recent meta-analysis involving patients with T2DM (96). One pathway through which ketogenesis may influence insulin sensitivity involves the hydroxycarboxylic acid receptor 2 (HCAR2), a member of the G-protein-coupled receptor (GPCR) family. HCAR2 plays a key role in anti-lipolytic and anti-inflammatory processes.

Zhang et al. demonstrated that BHB can activate HCAR2 and result in activated protein kinase A (PKA) pathway by increasing the intracellular calcium levels, which stimulates adenylate cyclase (AC) and increase cyclic AMP (cAMP) concentrations. Activated PKA inhibits Raf1 activity, leading to decreased ERK1/2 activity, inhibition of PPARγ Ser273 phosphorylation in adipocytes, and ultimately reduced insulin resistance (97). Additionally, BHB treatment was shown to increase AKT phosphorylation in mouse muscle (98). Ketogenesis can also exert positive effects on insulin sensitivity by regulating hepatic acyl-CoA level and TCA cycle (99). Moreover, by shuttling excess lipids into BHB, enhanced ketogenesis may promote hepatic insulin sensitivity (100).

## Gut microbiome dysbiosis in COVID-19 and implications for insulin resistance

Human observational studies have reported significant alterations in the fecal microbiome of COVID-19 patients compared to healthy individuals. These changes include: (1) decreased bacterial diversity (101, 102), a decrease in short-chain fatty acid (SCFA)-producing bacteria from the Eubacteriaceae, Ruminococcaceae, and Lachnospiraceae families and several gut commensals with known immunomodulatory potential such as Faecalibacterium prausnitzii, Eubacterium rectale and bifidobacteria. Concomitantly, there is an increased presence of opportunistic pathogens from the Enterobacteriaceae families (101–104). (2) An increase in the amount of opportunistic fungal pathogens, such as Aspergillus flavus, Candida albicans, and Candida auris (105). (3) A decrease of multiple bacteriophage lineages (DNA viruses) in the fecal of COVID-19 patients (106).

SCFA, which include acetate, propionate, and butyrate (107), play critical roles in energy metabolism and immune regulation. Previous studies have demonstrated that acetate administration activates AMP-activated protein kinase (AMPK) and promots fatty acid oxidation and increase energy expenditure (108). Furthermore, acetate infusion has been shown to elevate the levels of glucagon like peptide-1 (GLP-1) and peptide YY in the circulation of overweight women, thus modulating satiety (109). Similarly, butyrate administration can increase BAT mass and uncoupling protein-1 (UCP1) expression (110), which is shown to improve insulin sensitivity in obese mice treated with antibiotics (111).

SCFAs also exhibit anti-inflammatory properties, partly by inhibiting NF-κB activation in host immune cells through their interaction with GPR43 and GPR41 receptors (112, 113). Additionally, butyrate has been shown to promote the extrathymic generation of T regulatory (T(reg)) cells, which reduce macrophage infiltration in adipose tissue, a key factor in improving insulin sensitivity (114). The reduction of circulating SCFAs in COVID-19 patients may therefore play a significant role in the observed decrease in insulin sensitivity.

However, it should be acknowledged that the current understanding of the relationship between COVID-19-associated gut dysbiosis and post-COVID insulin resistance remains preliminary. Although the reduction in SCFA-producing bacteria and the metabolic and immunological functions of SCFAs have been reasonably described, direct clinical evidence linking specific post-COVID gut microbiome profiles to insulin resistance-related indicators within the same patient population is still limited. Most of the supporting evidence for the metabolic roles of SCFAs is derived from studies on obesity, metabolic syndrome, or general gut-metabolism crosstalk, rather than from cohorts specifically focusing on post-COVID conditions. Further targeted investigations in post-COVID patients are therefore needed to establish a more direct causal or associative link.

## Psychological impact of COVID-19 and Its association with insulin resistance

The COVID-19 pandemic has been strongly associated with psychological issues such as depression and anxiety (115), likely arising from factors such as fear of infection, social isolation, worsening economic conditions, and decreased serotonin (81, 116). In an Italian center, a survey found that during the lockdown period, 80% of children and adolescents reported experiencing psychological discomfort (33).

Despite the current paucity of studies that can directly demonstrate the exacerbation of insulin resistance by COVID-19 related psychological issues, a substantial body of prior research has firmly established the correlation between mental disease and insulin resistance. For example, a large meta-analysis reported increased insulin levels and HOMA-IR in individuals experiencing acute depression, with these levels not returning to normal during remission (117). Another epidemiological study suggested a link between insulin resistance and major depressive disorder (MDD) (118).

Investigative findings suggest that specific psychiatric disorders and insulin resistance may be underpinned by shared pathophysiological mechanisms. Genomic studies have identified shared polygenic risk factors between MDD and insulin resistance-related diseases, suggesting common underlying pathophysiological mechanisms (119). These mechanisms may include dysregulations in immune-inflammatory pathways, the hypothalamic-pituitary-adrenal axis, brain insulin signaling, and gut microbiota (120). Clinical studies reported that the pharmacotherapeutic agents utilized for the treatment of these two distinct conditions appear to exert ameliorative effects on the other condition as well. For instance, commonly prescribed antidepressants, specifically serotonin reuptake inhibitors (SSRIs), have shown benefits in improving insulin sensitivity in adults with comorbid T2DM and MDD, independent of weight loss or reduced glucose levels (120). Furthermore, certain oral hypoglycemic agents, such as PPAR-γ agonists, biguanides, and GLP-1 receptor agonists, have been tested as potential adjunctive therapies for depression. A meta-analysis of eight randomized controlled trials suggested that pioglitazone can improve depressive symptoms, particularly in females, regardless of their initial insulin resistance status (121).

## COVID-19 vaccines and insulin resistance

Previous studies have reported cases of life-threatening hyperglycemic crises occurring shortly after COVID-19 vaccination (122, 123). For instance, Edwards et al. (122) and Lee et al. (124) each reported three instances of hyperglycemic emergencies following administration of Pfizer-BioNTech (BNT162b2), Moderna (mRNA-1273), or ChAdOx1 nCoV-19-first dose respectively. These patients, with either preexisting or newly diagnosed T2DM, exhibited significant osmotic symptoms and elevated blood glucose levels, consistent with diagnoses of hyperosmolar hyperglycemic state (HHS) or HHS-DKA. Zilbermint et al. detailed a case of rapid-onset severe DKA and transient profound insulin resistance in a 24-year-old female with T1DM following the second dose of the mRNA-1273 messenger RNA COVID-19 vaccine (125). They subsequently conducted a prospective study to assess whether COVID-19 booster vaccines transiently exacerbated glycemic control and insulin resistance in individuals with T1DM. Among the 21 T1DM patients enrolled, mean daily glucose levels and insulin resistance (measured by total daily insulin resistance, TDIR) significantly increased at Days 2 and 3 post-vaccination (126). In a parallel investigation, Zhai et al. (127) conducted a prospective cohort study involving 180 participants to evaluate the impact of mRNA COVID-19 vaccine (BNT162b2) boosters on healthy controls, individuals with prediabetes, and patients with T2DM. After confirming no significant differences in immune response indices among the three groups, they observed heightened risks of glucose intolerance and insulin resistance in T2DM patients following booster shots, marked by substantial increases in HbA1c levels, HOMA-IR, and the triglyceride-glucose (TyG) index.

Zhai et al. (127) conducted an experiment to explore the mechanisms by which COVID-19 vaccination affects glucose metabolism. They administered the mRNA COVID-19 vaccine (BNT162b2) to healthy mice once a week for four consecutive weeks. The results showed that these mice developed impaired glucose tolerance and elevated C-peptide levels. Notably, glucose intolerance persisted in these mice for another 4-week even without further vaccination (at week 8). Consistent with the insulin tolerance test (ITT) findings, the level of insulin-induced Akt phosphorylation in insulin-sensitive tissues of these mice at week 8 was significantly reduced. Moreover, the NF-κB and MAPK signaling pathways in the liver tissues of these mice were markedly activated. In contrast, mice that received four doses of the inactivated COVID-19 vaccine (CoronaVac, Sinovac) did not exhibit significant impairments in glucose tolerance.

Preclinical and clinical studies have demonstrated a positive correlation between SARS-CoV-2 spike protein levels, induced by mRNA COVID-19 vaccination, and indices of glucose intolerance, insulin resistance, and serum TG levels. Zhai et al. (127) revealed that the SARS-CoV-2 spike protein directly inhibits insulin signaling *in vitro*, as shown by its dose- and time-dependent suppression of insulin-induced glucose uptake and phosphorylation of Akt and IRβ in 3T3-L1, HepG2, and C2C12 cell lines. They further showed that TLR4 blockade with antagonist IAXO-102 and estrogen receptor (ER) activation with agonist PPT significantly mitigated the SARS- CoV-2 spike protein’s inhibitory effects on insulin signaling in 3T3-L1 cells, indicating that the SARS- CoV-2 spike protein impairs insulin signaling via TLR4 and ER pathways. Consistent with these *in vitro* findings, ER activation or TLR4 inhibition effectively alleviated insulin sensitivity and glucose tolerance impairments induced by COVID-19 vaccination in mice. Indeed, studies have suggested that estrogen receptor modulates downstream TLR4 signaling molecules, such as MyD88 and TRIF, thereby dampening the inflammatory response (128). This crosstalk may reduce TLR4 signaling when the ER is activated.

Notebly, the SARS-CoV-2 spike protein may linger for several months post-COVID-19 vaccination, potentially influencing the onset of T2DM in susceptible individuals (129). However, these findings do not undermine the clinical efficacy of COVID-19 vaccines; rather, they underscore the necessity for enhanced monitoring of glycemic control in individuals scheduled to receive COVID-19 vaccine boosters.

## Dietary interventions for insulin resistance in COVID-19

Dietary interventions have been recommended for patients with post-COVID-19 syndrome (130). Here, we focus on dietary strategies that may improve insulin sensitivity in COVID-19 patients. Insulin resistance can manifest in liver and skeletal muscle. Recent studies have suggested that individuals with liver insulin resistance exhibit distinct metabolomic (131), lipidomic (132), adipose tissue transcriptomic, and systemic inflammatory profiles (133) compared to those with more pronounced muscle insulin resistance. As a result, these individuals may respond differently to dietary interventions. In the CORDIOPREV-DIAB study, a *post hoc* analysis showed that patients suffer from predominant muscle insulin resistance would respond better to a high monounsaturated fatty acids diet, while those with predominant liver insulin resistance responded more favorably to a high-complex carbohydrate, low-fat diet (134). Additionally, high-protein (135), high-fiber diets (136), and the Mediterranean diet (137) have been shown to reduce liver fat content, potentially improving hepatic insulin sensitivity (138). Moreover, the quality of dietary fat may specifically affect skeletal muscle lipid metabolism and peripheral insulin sensitivity (139).

## Therapeutic strategies for insulin resistance in COVID-19

### Metformin

Retrospective studies have shown that metformin-treated patients with T2DM exhibit reduced ICU admissions and mortality following SARS-CoV-2 infection compared to those not receiving metformin (140, 141). In recent years, metformin has been extensively investigated for its therapeutic potential in COVID-19 complications due to its metabolic control and potential anti-inflammatory and immune-regulating properties via multiple pathways (140–142). For instance, metformin enhances insulin sensitivity in COVID-19 patient by promoting glucose uptake and fatty acid oxidation through AMPK pathway activation (142). Additionally, metformin modulates immune function and inhibits excessive inflammation, such as by reducing levels of pro-inflammatory cytokines like IL-6 and TNF-α, thereby alleviating cytokine storm-induced insulin resistance (141, 142).

Interestingly, Zhai et al. (127) observed that insulin resistance was less impacted by COVID-19 mRNA vaccine boosters in diabetic patients receiving metformin during the study. They also demonstrated that metformin mitigated insulin resistance in mice treated with four doses of COVID-19 vaccine, as evidenced by improved OGTT and ITT indices and enhanced insulin signaling, without affecting serum levels of the SARS-CoV-2 spike protein. Consistent with these *in vivo* results, they further confirmed the beneficial effects of metformin on insulin sensitivity in 3T3-L1 cells, where metformin treatment significantly improved insulin signaling impaired by the SARS-CoV-2 spike protein. These findings suggest that metformin may serve as an adjuvant therapy to maintain glucose control during COVID-19 vaccination without significantly compromising immunogenic responses.

Notably, metformin may elevate the risk of lactic acidosis in patients with severe COVID-19, particularly in the presence of hypoxia or acute renal impairment, necessitating its discontinuation in such scenarios (140). In addition to metformin, glucagon-like peptide-1 (GLP-1) agonists and thiazolidinediones (glitazones) play a role in managing insulin sensitivity and supporting overall metabolic health in individuals with diabetes. Therefore, further prospective studies are warranted to elucidate the potential therapeutic value of these agents in addressing COVID-19-related insulin resistance.

### ACE2 enzymatic activators

Recent studies indicate that ACE2 may be a crucial link between SARS-CoV-2 infection and its metabolic consequences. Li et al. demonstrated that ACE2 knockdown in human umbilical vein endothelial cells results in increased expression of G6PC and decreased GLUT2, both of which are implicated in glucose metabolism. Conversely, ACE2 overexpression in obese mice markedly enhances insulin sensitivity and normalizes fasting glucose levels, nearly to the extent observed in lean mice. This improvement in glucose metabolism is evidenced by the downregulation of key glucose metabolism genes, including *G6pc, Pck1*, *Gpt1* and *Sglt1* (143). Moreover, ACE2 can mitigate inflammation by degrading pro-inflammatory Ang II and producing the protective peptide Ang-(1-7) (144).

Three ACE2 enzymatic activators—methazolamide, imatinib, and harpagoside—have demonstrated potential in ameliorating glucose and lipid metabolism following SARS-CoV-2 infection. In high-fat-diet-induced insulin-resistant mice, treatment with methazolamide or imatinib significantly enhanced glucose tolerance, insulin sensitivity, and normalized fasting glucose, insulin levels, and HOMA-IR. Additionally, these treatments reversed the expression of key metabolic genes: downregulated genes such as *Pgc1a* and *Glut2*, and upregulated genes like *G6pc, Pck1, Sglt1, Dgat2, Fasn*, and *Scd*, restoring them to levels comparable to those in lean mice (143). Similarly, Zhai et al. (127) illustrated that activating ACE2 using either the SARS-CoV-2 spike protein or the ACE2 enhancer resorcinol naphthalein could mitigate the insulin-desensitizing effects of the SARS-CoV-2 spike protein through alternative pathways. These findings collectively suggest that ACE2 activation may represent a promising therapeutic strategy for addressing COVID-19-induced insulin resistance.

Notably, the safety of ACE2 enzymatic activators is a subject of debate, primarily focusing on whether ACE2 activation increases the risk of viral entry into cells. Although ACE2 activation may theoretically enhance viral binding, most retrospective studies have not found that renin-angiotensin system (RAS) inhibitors (ACEI/ARB) exacerbate COVID-19 severity; instead, they may reduce mortality (145). Molecular dynamics simulations further indicate that ACE2 inhibitors (e.g., MLN-4760) can increase the affinity of the SARS-CoV-2 spike protein for ACE2 (146). Encouragingly, recent findings show that methazolamide, imatinib, and harpagoside not only activate ACE2 enzymatic activity but also directly inhibit viral entry into cells through allosteric inhibition of ACE2 binding to the SARS-CoV-2 spike protein (143). Recombinant human ACE2 (rhACE2) has also been shown to neutralize viral particles, reduce cell infection rates, and maintain enzyme activity to counteract Ang II-mediated damage (144, 147).

It is noteworthy that study associated with ACE2-based therapy remains in the preclinical or early translational phase, and its clinical applicability still awaits validation through population-based trials. Future research also should concentrate on developing methods for precisely regulating ACE2 activity to optimize the management of COVID-19-related metabolic disorders.

### Tocilizumab

Tocilizumab has a strong pathophysiological rationale for treating COVID-19. Several studies have demonstrated significant elevations in IL-6 levels among patients with severe COVID-19. By blocking the IL-6 signaling pathway, Tocilizumab can theoretically mitigate immune-mediated organ damage and metabolic disorders (148). Indeed, Montefusco et al. used Tocilizumab as an adjuvant therapy to reduce COVID-19-associated inflammation and found that patients with new-onset hyperglycemia treated with Tocilizumab exhibited greater reductions in glycemic levels at hospital discharge compared to those not receiving Tocilizumab (9).

However, it is important to note that Tocilizumab is predominantly utilized in the acute treatment of severe COVID-19 patients. Despite the established association between IL-6 and insulin resistance, Tocilizumab is not currently a viable therapeutic option for the management of metabolic dysfunction in long COVID. Future research endeavors are necessary to elucidate the clinical efficacy of Tocilizumab in insulin resistance, particularly in long COVID patients presenting with chronic IL-6 elevation.

### DPP-4 inhibitors

DPP-4, a molecule implicated in both metabolism and coronavirus infection, is commonly targeted by inhibitors used in the treatment of T2DM (149, 150). While SARS-CoV-2 primarily utilizes ACE2 for entry, structural modeling suggests potential interactions with DPP-4 as well (149). Elevated DPP-4 activity in individuals with metabolic syndrome has been associated with exacerbated inflammatory responses (149). Consequently, DPP-4 inhibitors may offer additional benefits through their anti-inflammatory properties and potential to interfere with viral entry, in addition to glycemic control (149, 150). However, the evidence for their role in post-COVID related insulin resistance is very limited. Further clinical study is required to substantiate these effects.

### Limitations

Several inherent and study-specific limitations must be acknowledged to contextualize the findings. First, methodological limitations inherent to narrative review design persist. Unlike systematic reviews with pre-specified protocols, registered in advance, and rigorous adherence to PRISMA guidelines, the literature selection and synthesis process in this review involved subjective judgment by the authors. Although comprehensive database searches were conducted, the narrative synthesis approach lacks standardized quantitative tools for evaluating study quality and synthesizing heterogeneous data, which may introduce interpretive bias in summarizing the mechanisms of COVID-19-induced insulin resistance. Additionally, publication and outcome reporting bias cannot be ignored. Published studies tend to prioritize positive findings, while negative results are underrepresented.

Second, heterogeneity in study populations and study designs limits the generalizability of conclusions. Included studies varied significantly in terms of participants’ characteristics, including age, comorbidities, disease severity, and COVID-19 variants. In addition, study designs of selected literature ranged from *in vitro* experiments, animal models, to clinical observational studies; discrepancies in experimental conditions, sample types, and detection methods further contributed to heterogeneity in the reported mechanistic data. Moreover, many mechanistic insights are derived from reliance on animal models.

Third, most of the supporting studies cited in some sections come from non-COVID populations. Because COVID-19 pandemic started in 2019 and the study of the post-COVID-19 syndrome still at its early stage, the analysis of the consequences of the many defects caused by SARS-CoV-2 infection still needs to learn from the previous studies of similar physiological conditions. For example, there is no direct evidence to substantiate that the unhealthy diet and less physical activity attributable to the COVID-19 lockdown engender the risk of insulin resistance. Our hypothesis that unhealthy diet and reduced physical activity may precipitate insulin resistance via adipokine dysregulation is extrapolated from investigations within the general population. Likewise, the effect of some of vitamin deficiency was mainly drawn from studies PCOS or T2DM. Similar situation is for the psychological impact to the development of insulin resistance. The discussion of psychological issue and insulin resistance drawn from general population studies but ont in long-COVID patients. In addition, although COVID-associated dysbiosis is clearly observed, however, whether this disturbed microbiota seen in COVID patient is the cause of post-COVID insulin resistance still need to be confirmed. While these studies support biological plausibility, they do not demonstrate that correcting these deficiencies or dysregulation in long-COVID patients improves insulin resistance.

Future research should aim to elucidate the long-term metabolic consequences of COVID-19, identify effective interventions such as dietary strategies and novel therapeutics, and develop personalized medicine approaches to address individual metabolic profiles.

## Conclusions

The COVID-19 pandemic has had a profound impact on global health, particularly in the realm of metabolic disorders, including insulin resistance and new-onset diabetes. This review has examined the multifactorial mechanisms underlying these metabolic complications, with a focus on the interplay between viral infection, immune dysregulation, lifestyle changes, and metabolic alterations. As illustrated in **Figure 1**, SARS-CoV-2 infection induces a pro-inflammatory response that impairs insulin signaling through pathways such as JAK/STAT, NF-κB, and TLR4. Concurrently, lockdown measures have led to reduced physical activity and unhealthy dietary habits, as well as deficiencies in vitamins and microelements due to decreased intake and absorption. These factors collectively exacerbate insulin resistance and increase the risk of obesity and type 2 diabetes. Psychological stress stemming from the pandemic has been linked to insulin resistance, underscoring the importance of mental health support. Additionally, imbalances in amino acid metabolism and impaired ketogenesis have been associated with insulin resistance in COVID-19 patients. Dysbiosis, characterized by reduced bacterial diversity and increased opportunistic pathogens, may also contribute to metabolic dysfunction. While COVID-19 vaccines are essential for controlling the pandemic, some may transiently affect glycemic control.

Therapeutic strategies can be considered to combat long-term effect caused by SARS-CoV-2 infection. Metformin has both metabolic and anti-inflammatory effects. Although ACE2-related strategies are still pre-clinical or early translational stage, it mechanistically interesting and worth anticipation. Lifestyle modification and dietary changes may also contribute to the comabting of long COVID syndrome. Future research should aim to elucidate the long-term metabolic consequences of COVID-19, identify effective interventions such as dietary strategies and novel therapeutics, and develop personalized medicine approaches to address individual metabolic profiles.

In conclusion, a comprehensive and multidisciplinary approach is crucial for addressing the metabolic challenges posed by COVID-19, with the goal of mitigating its impact and improving long-term health outcomes.
